# Supplementary material for: The interactome of intact mitochondria by cross-linking mass spectrometry provides evidence for coexisting respiratory supercomplexes
Source: Mol Cell Proteomics. 2017 Dec 8;17(2):216–32. doi: 10.1074/mcp.RA117.000470 (PMC5795388; doi:10.1074/mcp.RA117.000470)
Supplement: Supplemental Data [file supp_RA117.000470_133922_0_supp_23978_fzffwf.zip › spectra_annotation/mito_spectra_annotation/202-1-6-1-4-1.pdf]

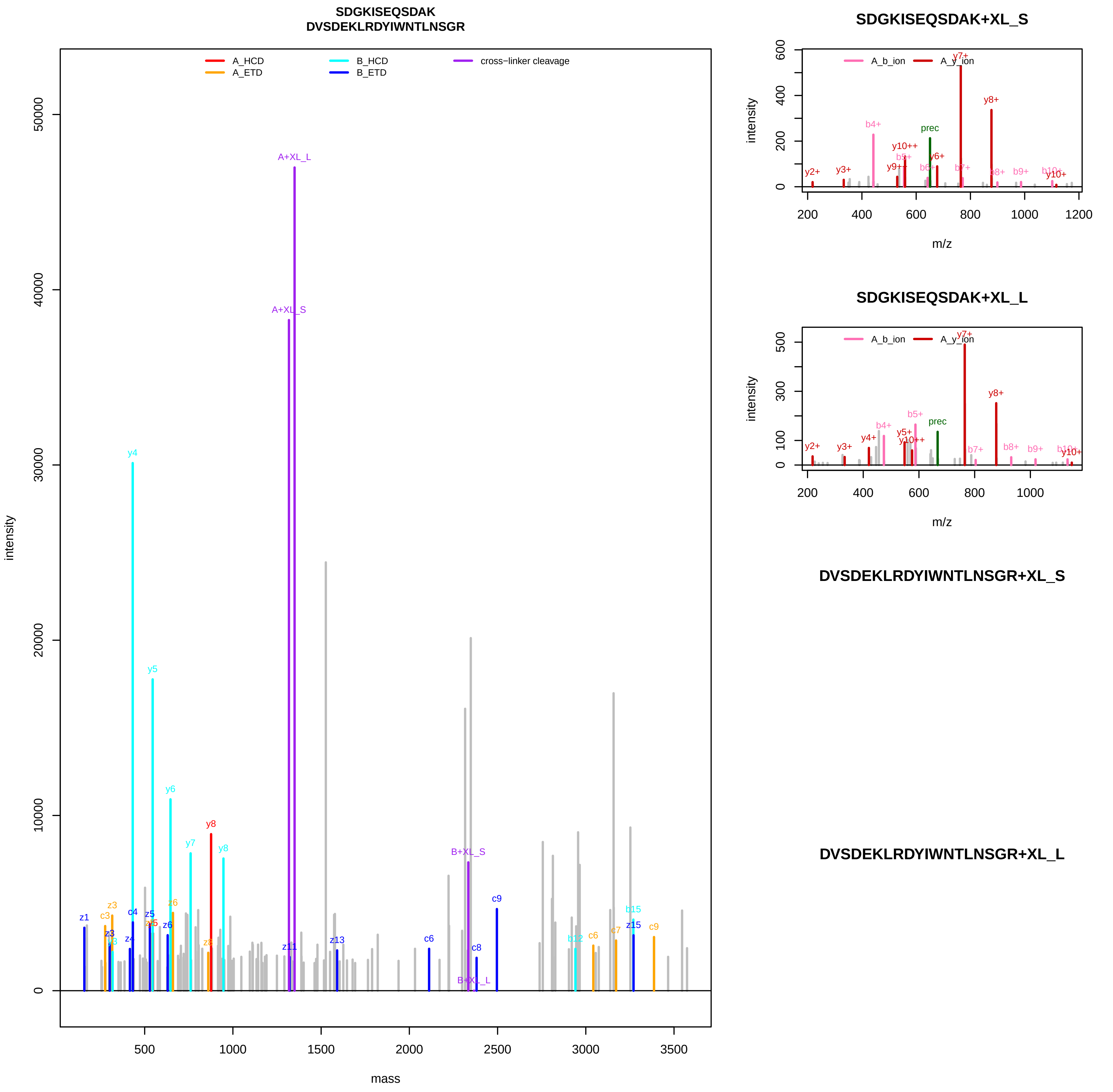

SDGKISEQSDAK
DVSDEKLRDYIWNTLNSGR
A_HCD
A_ETD
B_HCD
B_ETD
cross−linker cleavage
intensity
mass
0
10000
20000
30000
40000
50000
500
1000
1500
2000
2500
3000
3500
A+XL_L
A+XL_S
B+XL_S
B+XL_L
y4
y5
y6
y7
y8
z1
c3
z3
c4
z4
z5
z6
z8
z11
z13
c6
c8
c9
c7
b12
b15
z15
SDGKISEQSDAK+XL_S
A_b_ion
A_y_ion
m/z
prec
y2+
y3+
b4+
y9++
y10++
b5+
b6+
y6+
y7+
b7+
y8+
b8+
b9+
b10+
y10+
0
200
400
600
800
1000
1200
SDGKISEQSDAK+XL_L
y4+
y5+
100
300
500
DVSDEKLRDYIWNTLNSGR+XL_S
DVSDEKLRDYIWNTLNSGR+XL_L
